# Correlation between Malocclusions, Tonsillar Grading and Mallampati Modified Scale: A Retrospective Observational Study

**DOI:** 10.3390/children10061061

**Published:** 2023-06-14

**Authors:** Can Serif Kuskonmaz, Giovanni Bruno, Maria Lavinia Bartolucci, Michele Basilicata, Antonio Gracco, Alberto De Stefani

**Affiliations:** 1 Dental Clinic, Department of Neuroscience, University of Padua, 35121 Padova, Italyantonio.gracco@unipd.it (A.G.); 2 Department of Industrial Engineering, University of Tor Vergata, 00133 Roma, Italy; alberto.de.stefani@hotmail.it; 3 Section of Orthodontics, Department of Biomedical and Neuromotor Sciences, Alma Mater Studiorum—University of Bologna, 40126 Bologna, Italy; maria.bartolucci3@unibo.it; 4 UOSD Special Care Dentistry, Department of Experimental Medicine and Surgery, University of Rome Tor Vergata, 00133 Roma, Italy; michele.basilicata@ptvonline.it; 5 Department of Pharmacological Sciences, University of Padua, 35121 Padova, Italy

**Keywords:** tonsillar grading, tongue size, malocclusion, tonsil hyperplasia

## Abstract

Aim: to investigate the correlation between growth tendency and different patient malocclusion, tonsillar grading, and tongue size (Mallampati index). Materials and Methods: The sample is composed of 64 males and 40 females; patients aged between 6 and 16 years (median age 11 years, IQR 9–13) were included. The final sample is therefore 104 patients. After the first orthodontic visit, all the children underwent a collection of documentation (photographs, digital arch models, and X-rays). Patients were classified according to their malocclusion, palatal conformation, tonsillar grading, and Mallampati index. Group comparisons were performed using Fisher’s test. A *p*-value less than 0.05 was considered statistically significant. Results: The narrow palate was more frequent in those with Angle class III (*p* = 0.04), while the other variables considered (tonsillar grading, Mallampati, and lingual frenulum) were not significantly associated with dental class (*p* > 0.05). Furthermore, a different distribution of tonsillar grading was observed between subjects with normal palate and subjects with narrow palate, while no statistically significant association was found between the palatal shape and Mallampati index (*p* = 0.88). Conclusions: This study found that children with higher tonsillar grading had a higher prevalence of crossbite or narrow palate compared to other children at the same developmental stage. However, we did not observe any association between tonsil hyperplasia and the prevalence of class II malocclusion, anterior open bite, or patient divergence in this sample. Furthermore, no correlation was found between the Mallampati index and patients’ dental class, narrow palate, or divergence in this study.

## 1. Introduction

Malocclusion is a highly complex condition that is influenced by a multitude of factors. It is widely acknowledged as a multifactorial condition, meaning that it arises from the combined effects of various elements, including developmental factors, habits, genetic or hereditary factors, and ethnicity.

One notable developmental factor that can significantly contribute to the emergence of malocclusion is oral breathing. When an individual consistently breathes through their mouth rather than their nose, it can have profound implications for the growth and development of the jaws and facial bones. This is primarily due to the pivotal role played by the position and movement of the tongue during the breathing process, as they are crucial in maintaining the delicate balance of forces within the oral cavity and its surrounding structures. Consequently, any alterations in these forces can have a direct impact on the alignment of the teeth and the shape of the dental arches.

Mouth breathing can ultimately lead to the development of a high-arched palate, which is characterized by a narrow dental arch and teeth crowding. This occurs because the natural position of the tongue on the roof of the mouth is compromised, impeding the proper development of the upper jaw. The absence of the tongue’s upward pressure on the palate prevents it from fully expanding, resulting in a narrower arch and inadequate space for the teeth to erupt in their correct positions. Consequently, dental crowding becomes increasingly likely, as there is insufficient room for all the teeth to align harmoniously.

Moreover, mouth breathing can also result in changes in the position of the lower jaw, leading to a retruded or flattened appearance. The altered breathing pattern affects the function and posture of the facial muscles, which in turn influences the growth and development of the lower jaw. Consequently, the lower jaw may fail to achieve its optimal forward position, resulting in a retrusion or a flattened profile. These effects can further exacerbate the misalignment of the teeth and have a significant impact on the overall facial aesthetics.

Introducing optimal breathing patterns and maintaining proper oral health are crucial factors in promoting overall well-being in children. Among the various factors contributing to oral breathing in children, allergic rhinitis, adenoid hypertrophy, enlarged palatal tonsils, and obstructive nasal septal deviation stand out as key culprits [1]. However, it is widely recognized that hypertrophy of tonsils and adenoids is the most prevalent cause of upper airway obstruction in young children.

The implications of upper airway obstruction and altered breathing patterns on a child’s dentofacial growth cannot be overstated. Oral breathing often leads to a lower jaw position and, subsequently, a lower tongue position, which can have significant consequences for craniofacial growth and development [2]. Early recognition and intervention to address these issues are vital for promoting healthy facial growth and mitigating potential complications in the future.

To further emphasize the significance of tonsillar hypertrophy, an influential classification scale has been established by Brodsky and Koch [3]. This scale assesses the volume of the tonsils and the space they occupy between the anterior pillars of the oropharynx. By employing this scale, healthcare professionals are better equipped to evaluate the extent of tonsillar hypertrophy and customize appropriate interventions accordingly. This classification system plays a pivotal role in providing tailored treatment strategies and enhancing patient outcomes.

As researchers delve deeper into the intricate relationship between oral breathing, craniofacial growth, and the underlying causes such as allergic rhinitis, adenoid hypertrophy, enlarged palatal tonsils, and obstructive nasal septal deviation, the necessity of a multidisciplinary approach becomes increasingly evident. Collaborative efforts among pediatricians, otolaryngologists, dentists, orthodontists, and other healthcare providers are essential to effectively identify, diagnose, and treat these conditions [1,2,3,4,5].

Brodsky and Koch have developed a classification scale for tonsillar hypertrophy, which takes into account the volume of the tonsils and the space they occupy between the anterior pillars of the oropharynx [3]. This classification system consists of five grades, as detailed in Table 1.

In terms of assessing tongue size and relative volume within the oral cavity, the Mallampati index is widely utilized. Proposed by Seshagiri Mallamapati in 1985, this index aims to predict the likelihood of difficult intubations based on the degree of mouth opening. The scale ranks the visibility of the tonsillar pillars, uvula, and soft palate, thereby providing an indication of the airway view during direct laryngoscopy. Over time, some modifications have been made to the original scale, including the addition of a fourth class and the development of the Modified Mallampati Position (MMP). The MMP evaluates the oropharynx in its natural position without any protrusion or phonation.

The Mallampati index and its modifications are valuable tools in assessing airway characteristics, particularly in relation to intubation difficulty. These assessments help healthcare professionals anticipate potential challenges during intubation procedures and make informed decisions regarding the most appropriate techniques and interventions to employ.

In summary, the classification scale devised by Brodsky and Koch provides a standardized approach to evaluating tonsillar hypertrophy. Additionally, the Mallampati index and its variations serve as important tools in assessing airway characteristics and predicting intubation difficulty. By utilizing these tools, medical practitioners can enhance their understanding of patients’ anatomical features and tailor their treatment strategies accordingly. The Mallampati scale assesses the visibility of the tonsillar pillars, uvula, and soft palate to predict difficult intubations. The Mallampati modified scale expands on this by including the evaluation of the oropharynx in a natural position without protrusion or phonation, providing a more comprehensive assessment of airway visibility. The modified scale facilitates a more nuanced classification of airway visibility, improving the accuracy of predicting intubation difficulty [5]. The MMP classifies the visibility of the tonsils, pillars, uvula, and distal soft palate, and it currently exists as a four-class assessment tool (Table 2). 

This study aims to investigate the correlation between growth tendency and different patient malocclusions, tonsil grading, and tongue size. 

## 2. Materials and Methods 

The sample for this study consisted of 104 children who sought orthodontic evaluation at the Dental Clinic of Padua. Children who had previously undergone orthodontic treatments were excluded from the study. The sample included 64 males and 40 females (Table 3), with ages ranging from 6 to 16 years (median age 11 years, IQR 9–13). The final sample size amounted to 104 patients. To ensure the appropriate sample size for statistical analysis, a sample size calculation was performed, considering the desired level of significance, expected effect size, and statistical power. This calculation helped ensure that the sample size was sufficient to detect meaningful differences and provide robust findings.

The study design involved collecting comprehensive documentation from all the children following their initial orthodontic visit. This documentation encompassed intra and extraoral photographs, X-rays, and dental casts. Intraoral photographs comprised frontal, lateral, occlusal, frenulum, palatine tonsils, and resting tongue photographs. Extraoral photographs included frontal and profile views. All photographs were captured using the same camera (Nikon d7200, camera lens Nikkor Macro 105 mm. Nikon, Tokyo, Japan). Intraoral photographs were taken in manual mode with a focal length of 32, an exposure time of 1/125, and ISO 200. Extraoral photographs were taken in manual mode with a focal length of 8, an exposure time of 1/125, and ISO 200. Additionally, all patients underwent radiographic examinations, including orthopantomography and lateral cephalometric radiography for cephalometric measurements. The orthodontic evaluations were consistently conducted by the same operator. Cephalometric measurements included McLaughlin and Jarabak analysis. 

On each selected patient, the tonsil grade was determined by the same observer, who asked the patient to lie down in a supine position, open the mouth, and continuously pronounce the phoneme /r/. At the same time, the operator positioned a tongue depressor at the back of the tongue dorsum. The operator examined the pharynx without activating the vomiting reflex, rotating the tonsils to artificially bring them closer to the midline.

In the present study, various additional measurements were taken to complement the tonsil grading, MMP, skeletal and dental classification of patients, arch transversality, patient growth tendency (divergence) based on cephalometric measurements, and growth peak determined by vertebral measurements.

Furthermore, tongue size was assessed using a modified version of the Mallampati tongue position method. During the examination, patients were instructed to sit in a neutral head position and fully open their mouths. By employing a straightforward four-grade classification system that considers the visibility of the tonsillar pillars, uvula, and soft palate, Mallampati established a clear correlation between the grade of the Mallampati scale and the visual assessment of airway conditions during direct laryngoscopy.

This comprehensive approach facilitates a more comprehensive evaluation of patients, considering multiple factors such as tonsil grading, skeletal and dental class, arch transversality, growth tendencies, and tongue size. These assessments provide valuable insights into the anatomical characteristics and growth patterns of individuals, assisting healthcare professionals in formulating appropriate treatment strategies and optimizing patient care.

## 3. Statistical Analysis

Continuous data were expressed as median and interquartile range (IQR), while categorical data were expressed as frequency and percentage. Group comparisons were performed using Fisher’s test. A *p*-value less than 0.05 was considered statistically significant. Data analysis was conducted using R 4.2 (R Foundation for Statistical Computing, Vienna, Austria).

## 4. Results 

The study included a total of 104 participants, comprising 64 males and 40 females, ranging in age from 5 to 16 years (with a median age of 11 years and an interquartile range of 9–13 years). The characteristics of the subjects can be found summarized in Table 3. Table 4 presents the findings of the association investigation regarding dental class, indicating a higher prevalence of narrow palate in individuals with a dental class III (71% compared to 33–40% for classes I–II), approaching statistical significance with a *p*-value of 0.05. However, no significant associations were observed between dental class and the other variables examined, specifically tonsillar grading and MMP score.

In Table 5, the results of the association investigation with skeletal class are displayed, revealing a higher incidence of narrow palate among subjects with a skeletal class III (64% compared to 32–37% in classes I–II) with a statistically significant *p*-value of 0.04. Conversely, no significant associations were found between skeletal class and the other variables investigated, including tonsillar grading and MMP score.

The investigation of the association with divergence, as shown in Table 6, did not yield any statistically significant associations between the examined variables (tonsillar grading and Mallampati score) and divergence.

Furthermore, a significant disparity in tonsillar grading distribution was observed between subjects with a normal palate and those with a narrow palate (*p* = 0.007). However, no statistically significant association was detected between the palate type (normal or narrow) and Mallampati score (*p* = 0.88), as evidenced in Table 7.

## 5. Discussion

Recent years have witnessed extensive research on craniofacial changes in children with respiratory obstruction. However, the relationship between the cause of respiratory obstruction and its impact on craniofacial growth [4,5,6,7] has sparked significant debate in the literature. Among the various theories proposed, the prevailing view suggests that tonsil hypertrophy, leading to pharyngeal obstruction, results in oral breathing [8]. Consequently, children modify the positioning of their orofacial and jaw muscles, which, in turn, affects chewing, swallowing, and speech, ultimately leading to occlusal and skeletal alterations [9].

In the current study, findings reveal a significant association between tonsillar grade and a narrow palate: as the grade increases, the palate becomes narrower. This correlation is linked to the increased space occupied by the tonsils between the anterior pillars of the oropharynx. The natural growth and expansion of the maxilla are influenced by the forces exerted by the tongue, both at rest and during swallowing. It appears that optimal jaw expansion relies on the lateral forces applied by the tongue [9].

In the present study, it was observed that patients with grade 3 and 4 tonsillar hypertrophy were more likely to develop malocclusions compared to those with grades 0, 1, and 2.

Behlfelt et al. [10] demonstrated a significant and positive relationship between tonsil size and the depth of the palatine vault or narrowness of the maxillary arch. Other studies have also reported that enlarged tonsils leading to oral respiration can impede transverse growth of the maxilla and increase the prevalence of posterior crossbite [11,12].

Regarding anteroposterior size changes, both in the maxilla and mandible, no significant differences were observed, which aligns with findings from previous studies in the literature [13,14,15].

The role of a hyperdivergent facial morphology in Class II malocclusion as a risk factor for the development of Obstructive Sleep Apnea Syndrome (OSAS) remains unclear. It is uncertain whether it is a result of impaired growth response to OSAS or a potential predisposing factor for OSAS. The etiological connection between these morphological patterns and OSAS has not been definitively established.

The association between breathing patterns and postural changes, which forms the fundamental premise in linking Obstructive Sleep Apnea Syndrome (OSAS) and craniofacial morphology, lacks conclusive evidence. The presence of a habitual open-mouth posture in cases of benign lip incompetence does not necessarily indicate a predominantly oral breathing pattern. Interestingly, our findings contradict certain studies [16,17,18] that have reported correlations between upper airway characteristics and malocclusion type, revealing a narrower nasopharynx in individuals with Class II malocclusion.

A recent study by Festa et al. further supports our findings, demonstrating a high prevalence of orthodontic problems in mouth-breathing children. Severe tonsillar hypertrophy is implicated in the presence of malocclusion and increased overjet. Additionally, the association between mild tonsillar hypertrophy and various occlusal anomalies in mouth-breathers suggests the significant role of malocclusion in initiating oral breathing patterns in children [19].

While oral breathing likely contributes to the etiology of such malocclusions, it is crucial to recognize that heredity plays a larger role in facial growth and development. Consequently, it is not always possible to anticipate the occurrence of dental abnormalities in individual cases. Nonetheless, epidemiologically, mouth-breathing children exhibit a higher risk of developing Class II malocclusion, anterior open bite, and posterior crossbite compared to the general population, as demonstrated by other studies.

It is imperative to continue investigating the complex interplay between breathing patterns, craniofacial morphology, and malocclusion development to gain a comprehensive understanding of their associations. Further research is needed to elucidate the multifactorial nature of these conditions and establish effective preventive and treatment strategies. In the context of preventing and treating craniofacial growth disorders, it is crucial to address and correct bad habits and mouth breathing, as they are recognized risk factors for malocclusion. Early interception and intervention can help prevent the development or exacerbation of malocclusions. Therefore, it is essential to adopt a multidisciplinary approach when managing patients in this regard [20].

While the current study contributes valuable insights, it is important to acknowledge its limitations. One potential limitation is the possibility of inter-operator variability in head positioning during the acquisition of intraoral photographs for the MMP assessment. This variability could potentially impact the accuracy and consistency of the collected data. To mitigate this limitation, standardized protocols and rigorous training can be implemented to ensure consistent and reliable measurements.

Another limitation is the nature of our sample, which consisted of patients seeking orthodontic evaluation at a university clinic. This sample may not be fully representative of the general population or even the orthodontic population in private practices. Therefore, caution should be exercised when generalizing our findings to broader populations. Future studies could consider incorporating larger and more diverse samples to enhance the generalizability of the results.

Despite these limitations, the present study provides valuable insights into the association between respiratory obstruction, craniofacial growth, and malocclusion. It emphasizes the importance of early identification and intervention to mitigate the potential negative impacts on craniofacial development. By addressing these issues promptly, healthcare professionals can contribute to improved patient outcomes and long-term oral health. Further research is warranted to expand our understanding of these relationships and explore effective preventive and treatment strategies in a broader context. 

## 6. Conclusions

This study revealed a significant association between higher tonsillar grading and an increased occurrence of crossbite or narrow palate among children at a similar stage of development. However, no significant associations were found between tonsil hyperplasia and the prevalence of class II malocclusion, anterior open bite, or patient divergence in this sample. Additionally, the Mallampati index did not show any correlation with patients’ dental class, narrow palate, or divergence in this study. It is worth noting that these findings are specific to the examined sample and may not be generalized to the wider population. Further research is warranted to explore these relationships in a more diverse population.

## Figures and Tables

**Table 1:** Palatine tonsil hypertrophy classification.

Brodsky and Koch Classification		
Grade	% Occupied	Definition
Grade 0	tonsils within the tonsil fossa	Tonsillectomy
Grade 1	tonsils occupy ≤ 25% of the oropharyngeal width	Intravelic atrophic tonsils
Grade 2	tonsils occupy 25–50% of the oropharyngeal width	Barely visible tonsils protruding minimally from the anterior pillars
Grade 3	tonsils occupy 50–75% of the oropharyngeal width	Hypertrophic tonsils occupying a space equal to 3/4 of the isthmus of the fauces
Grade 4	tonsils occupy >75% of the oropharyngeal width	Tonsils completely obstructing the isthmus of the fauces (kissing tonsils)

**Table 2:** Tongue size classification according to modified Mallampati index (MMP).

MMP	
Class	Definition
Class I	visualization of the tonsils, pillars, and entire uvula
Class II	visualization of the uvula but not the tonsils or the pillars
Class III	visualization of part of the soft palate but not the tonsils, pillars, distal soft palate, or base of the uvula
Class IV	visualization of the hard palate only

**Table 3:** Characteristics of the study sample.

	N	104
	Male	64 (62%)
	Age: Median (IQR)	11 (9–13)
Dental Class	I	36/103 (35%)
	II	53/103 (51%)
	III	14/103 (14%)
Skeletal Class	I	31 (30%)
	II	48 (46%)
	III	25 (24%)
Divergence	Hyperdivergent	35 (34%)
	Hypodivergent	24 (23%)
	Normodivergent	45 (43%)
Palatal Tonsil Grading	0	3 (3%)
	I	47 (45%)
	II	31 (30%)
	III	22 (21%)
	IV	1 (1%)
Modified Mallampati Index	I	5 (5%)
	II	59 (57%)
	III	40 (38%)
	IV	0
Arch Transversality	Normal	60 (58%)
	Narrow	44 (42%)

IQR: interquartile range.

**Table 4:** Association with the dental class. * is significant *p*-value.

	Dental Class I	Dental Class II	Dental Class III	*p*-Value
Tonsillar Grading				0.46
0	2 (6%)	1 (2%)	0 (0%)	
I	20 (55%)	23 (43%)	4 (29%)	
II	9 (25%)	15 (28%)	7 (50%)	
III	5 (14%)	13 (25%)	3 (21%)	
IV	0 (0%)	1 (2%)	0 (0%)	
Mallampati				0.2
I	2 (6%)	3 (6%)	0 (0%)	
II	25 (69%)	27 (51%)	6 (43%)	
III	9 (25%)	23 (43%)	8 (57%)	
IV	0	0	0	
Arch Transversality				0.05 *
Normal	24 (67%)	32 (60%)	4 (29%)	
Narrow	12 (33%)	21 (40%)	10 (71%)	

**Table 5:** Association with the skeletal class. * is significant *p*-value.

	Skeletal Class I	Skeletal Class II	Skeletal Class III	*p*-Value
Tonsil Grading				0.94
0	0 (0%)	2 (4%)	1 (4%)	
I	16 (52%)	20 (42%)	11 (44%)	
II	8 (26%)	14 (29%)	9 (36%)	
III	7 (22%)	11 (23%)	4 (16%)	
IV	0 (0%)	1 (2%)	0 (0%)	
Mallampati				0.81
I	1 (3%)	3 (6%)	1 (4%)	
II	18 (58%)	29 (61%)	12 (48%)	
III	12 (39%)	16 (33%)	12 (48%)	
IV	0	0	0	
Palate				0.04 *
Normal	21 (68%)	30 (63%)	9 (36%)	
Narrow	10 (32%)	18 (37%)	16 (64%)	

**Table 6:** Association with divergence.

	Hyperdivergent	Hypodivergent	Normodivergent	*p*-Value
Tonsil Grading				0.93
0	1 (3%)	1 (4%)	1 (2%)	
I	17 (48%)	11 (46%)	19 (42%)	
II	8 (23%)	7 (29%)	16 (36%)	
III	8 (23%)	5 (21%)	9 (20%)	
IV	1 (3%)	0 (0%)	0 (0%)	
Mallampati				0.8
I	2 (6%)	0 (0%)	3 (7%)	
II	20 (57%)	13 (54%)	26 (58%)	
III	13 (37%)	11 (46%)	16 (35%)	
IV	0	0	0	
Arch Transversality				0.64
Normal	18 (51%)	14 (58%)	28 (62%)	
Narrow	17 (49%)	10 (42%)	17 (38%)	

**Table 7:** Association with the arch transversality. * is significant *p*-value.

	Normal	Narrow	*p*-Value
Tonsil Grading			0.007 *
0	3 (5%)	0 (0%)	
I	33 (55%)	14 (32%)	
II	16 (27%)	15 (34%)	
III	7 (11%)	15 (34%)	
IV	1 (2%)	0 (0%)	
Mallampati			0.88
I	3 (5%)	2 (5%)	
II	35 (58%)	24 (54%)	
III	22 (37%)	18 (41%)	
